# High-affinity RNA binding by a hyperthermophilic single-stranded DNA-binding protein

**DOI:** 10.1007/s00792-016-0910-2

**Published:** 2017-01-10

**Authors:** Michael J. Morten, Roland Gamsjaeger, Liza Cubeddu, Ruvini Kariawasam, Jose Peregrina, J. Carlos Penedo, Malcolm F. White

**Affiliations:** 1 Biomedical Sciences Research Complex, University of St Andrews, St Andrews, KY16 9ST UK; 2 School of Science and Health, Western Sydney University, Locked Bag 1797, Penrith, NSW 2751 Australia; 3 School of Molecular Bioscience, University of Sydney, Sydney, NSW 2006 Australia

**Keywords:** RNA-binding proteins, OB fold, Single-molecule dynamics, Förster resonance energy transger, Nuclear magnetic resonance

## Abstract

Single-stranded DNA-binding proteins (SSBs), including replication protein A (RPA) in eukaryotes, play a central role in DNA replication, recombination, and repair. SSBs utilise an oligonucleotide/oligosaccharide-binding (OB) fold domain to bind DNA, and typically oligomerise in solution to bring multiple OB fold domains together in the functional SSB. SSBs from hyperthermophilic crenarchaea, such as *Sulfolobus solfataricus*, have an unusual structure with a single OB fold coupled to a flexible C-terminal tail. The OB fold resembles those in RPA, whilst the tail is reminiscent of bacterial SSBs and mediates interaction with other proteins. One paradigm in the field is that SSBs bind specifically to ssDNA and much less strongly to RNA, ensuring that their functions are restricted to DNA metabolism. Here, we use a combination of biochemical and biophysical approaches to demonstrate that the binding properties of *S. solfataricus* SSB are essentially identical for ssDNA and ssRNA. These features may represent an adaptation to a hyperthermophilic lifestyle, where DNA and RNA damage is a more frequent event.

## Introduction

Single-stranded DNA-binding proteins (SSBs) are essential for the genome maintenance of all known cellular organisms (Mushegian and Koonin 1996; Ashton et al. 2013) and are present in many viruses (Sun and Shamoo 2003). They play a vital role in DNA metabolism (Dickey et al. 2013), sequestering and protecting transiently formed ssDNA during DNA replication and recombination, melting double-stranded DNA (dsDNA), and detecting DNA damage and recruiting repair proteins (Ashton et al. 2013; Sun and Shamoo 2003; Dickey et al. 2013; Theobald et al. 2003; Suck 1997). SSBs from the three domains of life share little sequence similarity and diverse subunit organisation (Dickey et al. 2013), but a common evolutionary feature of the SSB protein family is the oligonucleotide/oligosaccharide-binding (OB) fold (five-stranded beta-sheet coiled to form a closed beta-barrel), which can bind ssDNA with high affinity (Theobald et al. 2003). Although the persistence of the OB fold in all SSBs suggests a common ancestor for these proteins (Suck 1997), the organisation of OB folds in SSBs varies considerably (Theobald et al. 2003). For example, *Escherichia coli* SSB (*Eco*SSB) is a homotetramer, with each subunit consisting of a single OB domain for ssDNA binding, in conjunction with a flexible C-terminal extension involved in protein–protein interactions (Raghunathan et al. 2000). The *Deinococcus*/*Thermus* SSBs, whilst still utilising the tetrameric functional binding mode, arrive at this arrangement by combining two SSB homodimers: each SSB monomer encoding two OB folds linked by a conserved spacer sequence (Dabrowski et al. 2002). All eukaryotes utilise a heterotrimeric SSB known as replication protein A (RPA) with six OB folds; two that mediate subunit interaction and four that are involved in ssDNA binding (Theobald et al. 2003; Bochkarev et al. 1999), whilst many also encode a second SSB (hSSB1/NABP2/OBFC2B) with a single OB fold, which is involved in the maintenance of genome stability (Richard et al. 2008; Wu et al. 2016).

The arrangement of euryarchaeal SSBs is similar to eukaryotic RPA: a polypeptide or polypeptides with multiple OB folds, including a characteristic OB fold interrupted by a zinc-binding domain (White 2003; Komori and Ishino 2001). This zinc-domain is also found in the large RPA70 subunit in eukaryotic RPA. It appears that some euryarchaeal SSBs form heterotrimers and others heterodimers (Komori and Ishino 2001). In contrast, the crenarchaeal SSB has a bacterial-like domain structure, with a single OB fold followed by a flexible C-terminal tail that is not involved in DNA binding and coats ssDNA with a stoichiometry of approximately 5 nucleotides (nt) DNA per SSB molecule (Wadsworth and White 2001). The crystal structure of the OB fold of *Sulfolobus solfataricus* SSB (*Sso*SSB) demonstrated its close structural relationship with the ssDNA-binding domains of human RPA70 (Kerr et al. 2003) and that of hSSB1 (Touma et al. 2016). The monomeric structure of SsoSSB in solution, both in the absence and presence of ssDNA, was recently confirmed by cross-linking experiments (Gamsjaeger et al. 2015) and by EPR and single-molecule molecule FRET (Morten et al. 2015).

Organisms inhabiting extreme environments where DNA damage is more frequent have a particular need to protect ssDNA, which is much more sensitive to damage than dsDNA (Ashton et al. 2013; Dickey et al. 2013). For example, the bacterium *Deinococcus radiodurans* maintains a high level of SSB in the cell and increases that level nearly three-fold in response to ionising radiation (Bernstein et al. 2004). Likewise, hyperthermophilic organisms, such as *S. solfataricus*, are also likely to experience elevated levels of DNA damage and it has been shown that *Sso*SSB expression is stimulated after UV irradiation (Wadsworth and White 2001; Gotz et al. 2007). Mutants of the archaeal halophile *Halobacterium* sp NRC1 with enhanced resistance to ionising radiation were shown to have enhanced expression of RPA (DeVeaux et al. 2007). *E. coli*, on the other hand, maintains a constant level of SSB that does not increase significantly in response to any DNA damage (Meyer and Laine 1990).

## Materials and methods

### Protein expression and purification

Recombinant SSB from *S. solfataricus* was prepared and purified as described previously (Wadsworth and White 2001). The A114C variant was constructed by site directed mutagenesis using standard protocols (QuikChange, Stratagene) and the sequences of oligonucleotides used for cloning and mutagenesis are available upon request. The variant was purified in the same manner as the wild-type *Sso*SSB. The A114C variant was then labelled with Alexa Fluor 647 using the manufacturers labelling buffer (Life Technologies) and a ten times molar excess of fluorescent dye with the addition of urea to a final concentration of 8 M. The labelling reaction was left at room temperature for 3 h, and then overnight at 4 °C. The labelling mixture was then diluted with labelling buffer to half the concentration of urea. A pure sample of labelled proteins was obtained using an affinity column to remove the unlabelled proteins and any remaining free dye, as described previously (Wadsworth and White 2001). The labelling efficiency was checked by UV–vis spectroscopy and MALDI-TOF mass spectrometry and found to be >90%.

### Oligonucleotides

Oligonucleotides were purchased from Eurofins MWG Operon and Qiagen. Sequences of oligonucleotides used in this study are shown in the table below. The positions of introduced biotin, fluorescein (FAM), and Cy3 and Cy5 dyes are indicated.

**Table:** 

Name	Sequences (5′–3′)
R21U	UUUUUUUUUUUUUUUUUUUUU
R21A	AAAAAAAAAAAAAAAAAAAAA
RNA-FAM	FAM-UGAUAAUCUCUUAUAGAAUUGAAAG
C12ssDNA	Biotin-CCCCCCCCCCCC-Cy3
C12ssRNA	Biotin-rCCCCCCCCCCCC-Cy3
RNA Hairpin	Cy5-rUGAUAAUCUCUUAUAGAAUUGAAAGU-Cy3

### Isothermal titration calorimetry (ITC)

Calorimetric experiments were carried out using a VP-ITC instrument (MicroCal). All solutions were degassed prior to use. *Sso*SSB samples were dialysed extensively against 20 mM MES buffer, pH 6.5, 100 mM potassium glutamate, and 1 mM MgCl_2_ (ITC buffer). Oligonucleotides (DNA or RNA) were also dissolved in ITC buffer. Binding experiments were performed in triplicate at 50 °C. A 370 µL syringe with stirring at 300 rpm was used to titrate the oligonucleotide (40 µM) into the sample cell containing approximately 1.4 mL of *Sso*SSB (10 µM). Each titration consisted of a preliminary 1 µL injection followed by up to 25 subsequent 10 µL injections. Heats of dilution (ΔH) were measured in corresponding blank titrations by adding oligonucleotide to ITC buffer and/or ITC buffer to protein and were found to be similar to heats observed at the end of protein-DNA titrations. ITC-binding isotherms were analysed using a Single Set of Identical Sites model built-in to ITC Data Analysis in ORIGIN provided by the manufacturer. Non-linear least-squares fitting of the data to this model was performed using the ITC Data Analysis software. This fit does not consider any positive cooperativity and the *K*_D_ values obtained are thus reported as “apparent *K*_D_’s”. This does not affect the main observation which is that RNA and DNA are bound similarly.

### Ensemble-fluorescence experiments

Protein-induced fluorescence enhancement (PIFE) experiments were carried out in triplicate using a Varian Cary Eclipse fluorimeter, exciting the Cy3 dye at 550 nm. Oligonucleotides C12ssDNA Cy3 and C12ssRNA Cy3 (10 nM) were solubilized in 50 mM Tris–HCl pH 8.0, 10 mM KCl, and titrated with *Sso*SSB in the same buffer. Emission intensity at each concentration of *Sso*SSB was corrected for dilution and the emission titration was fitted, as previously described (Morten et al. 2015), to a Hill model using Eq. 1.

1 $$\frac{{{F}_{\text{SSB}}}}{{{F}_{\text{o}}}}=\frac{{{B}_{\text{max}}}{{X}^{n}}}{\left( K_{\text{D}}^{n}+{{X}^{n}} \right)}$$

where *B*_max_ represents the maximum specific binding, *K*_D_ is the concentration required for half-maximum binding, and *n* is the Hill coefficient.

Melting experiments were carried out using and intra-molecular FRET assay using Cy3 and Cy5 as FRET pair and the energy transfer efficiency was calculated using Eq. 2 and transformed into unwound fraction of hairpin. In Eq. 2, *I*_D_^A^ and *I*_D_ represent the intensity of the donor in the presence and absence of acceptor, respectively. Control experiments to determine the variation in the emission of Cy3 due to PIFE at each *Sso*SSB were also carried out.

2 $${{E}_{\text{FRET}}}=1-\frac{I_{\text{D}}^{\text{A}}}{{{I}_{\text{D}}}}.$$

Stoichiometric tryptophan quenching experiments were carried out as previously described (Ashton et al. 2013). We used an excitation wavelength of 300 nm and we titrated a 10 nM solution of unlabelled *Sso*SSB with increasing concentrations of unlabelled oligonucleotide. The area under the emission spectrum was taken at each data point. All ensemble data shown represent the average of three replicates.

### Single-molecule fluorescence

Single-molecule FRET data were taken using a home-built single-molecule prism-type total-internal reflection microscope. Surface-immobilized oligonucleotides labelled with a donor Cy3 dye were exposed to *Sso*SSB labelled with the acceptor dye Alexa 647 as previously described (Morten et al. 2015). Quartz slides were passivated using a PEG surface and biotin/neutravidin interactions head groups were exploited to immobilise C12 ssDNA Cy3 and C12 RNA Cy3 (Blouin et al. 2009). The sample was excited by a 532 nm laser (Crystalaser, USA) and the fluorescence from the donor and acceptor was collected using an electron-multiplying CCD camera (Ixon, Andor). Single-molecule intensity traces were analysed using laboratory-written MATLAB routines as previously described (McCluskey et al. 2013). Apparent FRET efficiencies after background corrections were calculated using (*I*_A_/(*I*_A_ + *I*_D_)), where *I*_A_ and *I*_D_ represent the intensities of the acceptor and donor, respectively. Single-molecule FRET histograms were generated using the first 15 frames of each trajectory as previously reported (Morten et al. 2015; Bluoin et al. 2009; McCluskey et al. 2013). Single-molecule dwell-time histograms were calculated manually after filtering for blinking and photobleaching effects and fitted to a monoexponential decay curve to extract the corresponding transition rate. Measurements were carried out at room temperature with integration times of 50 ms per frame. The imaging buffer was identical to the ensemble binding buffer, with 200 μM Trolox, 6% (w/w) glucose and 0.1 mg/mL glucose oxidase, and 0.02 mg/mL glucose catalase added to reduce the rate of photobleaching and blinking of the fluorescent dyes.

### NMR experiments and modelling

NMR HSQC experiments were carried out using 0.8–1 mM *Sso*SSB OB domain (1–114) (Gamsjaeger et al. 2015) in the presence and absence of equimolar amounts of ssDNA (6T) and RNA (6U) (purchased from Sigma Aldrich), respectively, at 298 K on a Bruker 600 MHz spectrometer (Bruker Advance III) equipped with 5-mm TCI cryoprobes. An in-silico model was calculated using HADDOCK (Dominguez et al. 2003; de Vries et al. 2007) using the NMR structure (PDB ID 2MNA) as a template (Gamsjaeger et al. 2015). DNA (6T) was replaced by RNA (6U) and the definition of semiflexible and flexible residues; all ambiguous and unambiguous interaction restraints (AIR and UIRs, respectively) as well as base planarity restraints were taken from the docking calculations of the *Sso*SSB-DNA structure (Gamsjaeger et al. 2015).

### Exosome protection assay

*Sulfolobus solfataricus* exosome was purified as described previously (Witharana et al. 2012). 200 nM RNA labelled with a 5′-fluorescein (RNA-FAM) was incubated with wild-type SSB (concentrations from 0 to 480 µM) for 5 min at room temperature in 20 mM HEPES (pH 7.9), 0.1 mM EDTA, 60 mM KCl, 8 mM MgCl_2_, 2 mM DTT, and 10 mM K_2_HPO_4_. To each aliquot, 0.5 µl *S. solfataricus* Rrp41-Rrp42 hexameric ring and 0.4 µl Rrp4 protein were added. The total volume of each aliquot was 10 µl. The reaction was left to incubate at 60 °C for 1 h. 10 µl of each sample was added to acid phenol (Ambion) and mixed thoroughly, then spun at 13,000 rpm for 1 min. 5 µl from the resulting supernatant was added to 5 µl formamide (Promega) and loaded on to a denaturing gel (25% polyacrylamide, 7 M urea, 300 µl of ammonium persulfate (APS), and 30 µl of TEMED, 5 ml TBE, 5 ml water, total volume 50 ml) run at 85 W with a temperature threshold of 50 °C for 2.5 h. The gel was scanned using Fuji FLA5000 phosphorimager and analysed using the ImageJ software.

## Results and discussion

**Fig. 1:**
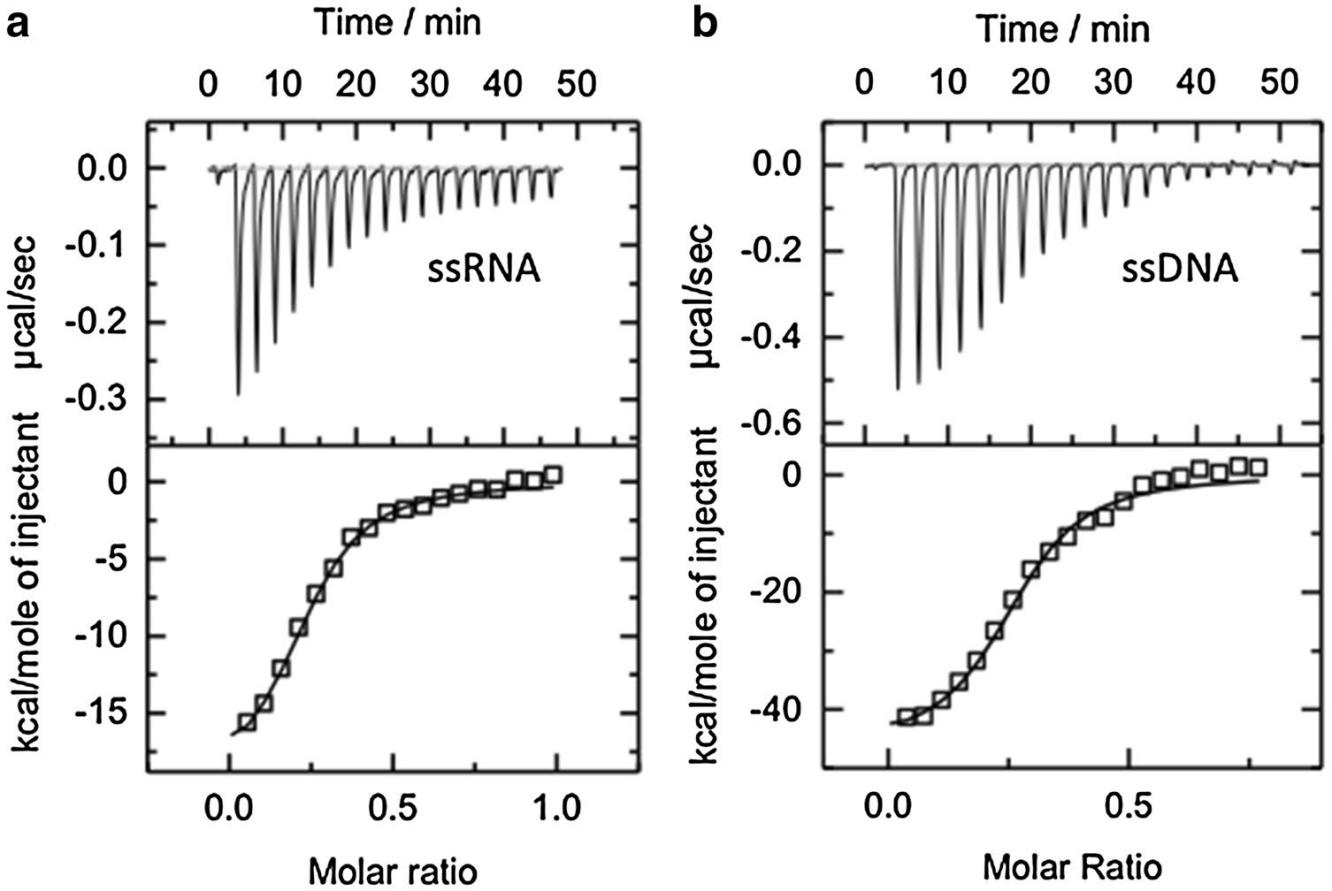
Representative isothermal titration calorimetry profiles for the interaction of *Sso*SSB with a 21 nt poly-A DNA oligonucleotide (**a**) and a 21 nt poly-rA RNA oligonucleotide (**b**). The *top panel* shows heat differences obtained for injections of 40 µM ssDNA or ssRNA into 10 µM *Sso*SSB solution. Titrations were completed in triplicate. The *lower panel* shows the incremental enthalpy changes, corrected for heats of dilution, with experimental data points (*open square*) and the best fit (*solid line*). ITC-binding isotherms were analysed using a single set of identical sites model in microcal origin

The previous studies of eukaryotic and bacterial SSBs have suggested efficient discrimination between ssDNA and RNA (Mushegian and Koonin 1996; Ashton et al. 2013). In mesophilic organisms, this discrimination may serve to ensure that SSB is reserved for binding to ssDNA during replication and repair, not distributed over the much more abundant and omnipresent RNA in the cell. The ability of these proteins to discriminate between ssDNA and ssRNA is not entirely understood, but it is thought to result from a combination of factors, including the lower plasticity of the RNA sugar pucker and the steric clash due to the presence of the 2′ hydroxyl group that increases the energy barrier for binding and limits the conformational landscape of ssRNA (Shamoo 2002). Usually, SSB proteins have only modest affinities for ssRNA (Meyer and Laine 1990). For instance, human RPA binds to ssRNA with an affinity at least three orders of magnitude lower than that for binding ssDNA (Kim et al. 1992) and the early studies on the *Escherichia coli* SSB also indicated a much weaker affinity to ribopolymers than to their deoxy-counterparts (Ruyechan and Wetmur 1976; Molineux et al. 1975). Bacterial cold shock proteins have been also reported to exhibit more than one order of magnitude decrease in binding affinity to ssRNA compared to ssDNA (Sachs et al. 2012). We were, therefore, surprised to observe using isothermal titration calorimetry that *Sso*SSB binds to a 21U RNA oligonucleotide (Fig. 1a) with a similar affinity (apparent *K*_D_ = 93 ± 0.4 nM) as that seen for a 21T DNA oligonucleotide (apparent *K*_D_ = 95 ± 0.6 nM) (Fig. 1b).

**Fig. 2:**
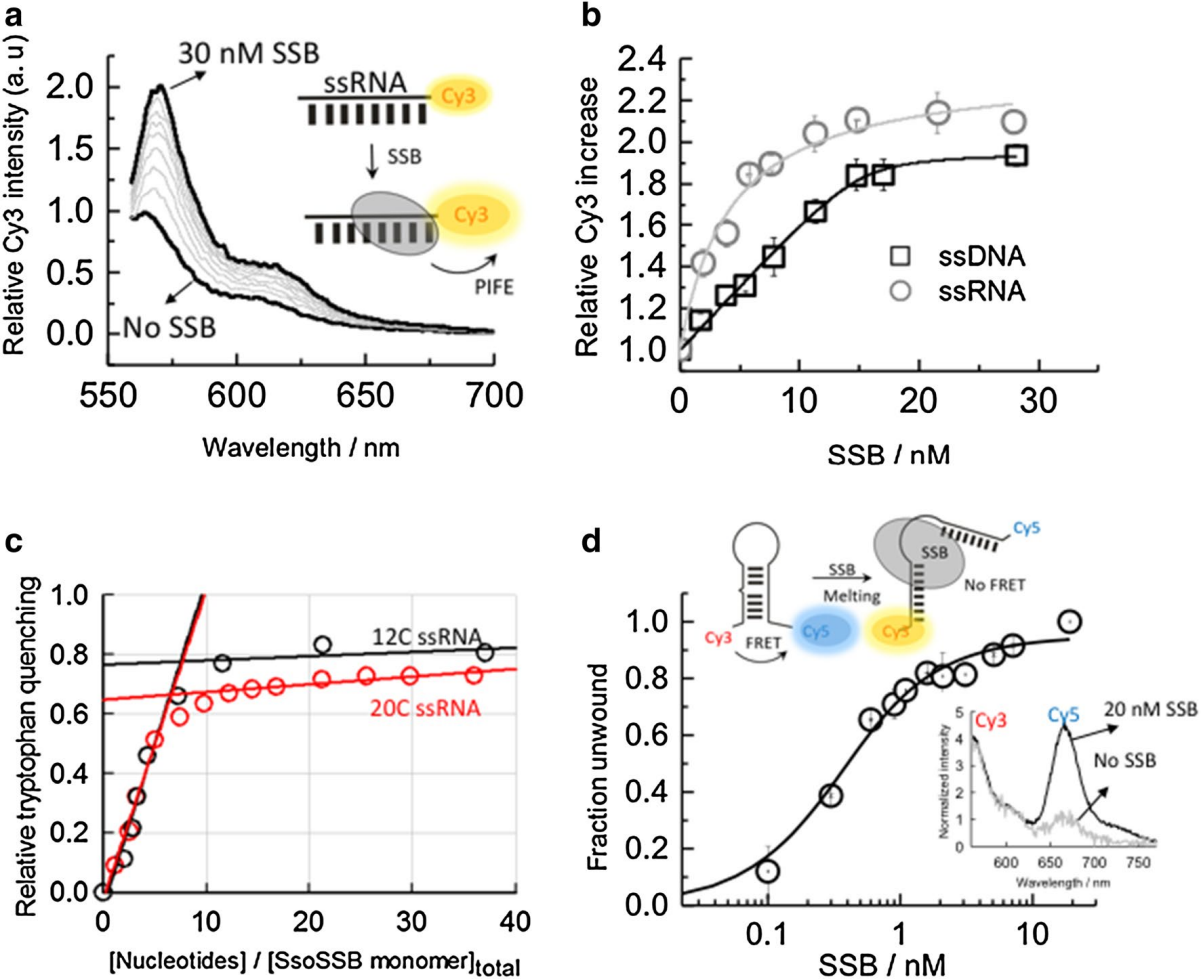
Ensemble-fluorescence characterization of the *Sso*SSB interaction with single-stranded RNA oligonucleotides. **a** *Sso*SSB binding to a 12-mer single-strand Cy3-labelled RNA monitored using protein-induced fluorescence enhancement (PIFE). Fluorescence emission spectra of Cy3 as a function of *Sso*SSB concentration. The fluorescence spectrum in the absence of *Sso*SSB was normalized to unity at the wavelength of the maximum and taken as a reference to calculate the emission enhancement at each *Sso*SSB concentration. **b** Relative variation in the emission intensity of a Cy3-labelled 12-mer ssDNA (*black squares*) and a Cy3-labelled 12-mer ssRNA (*grey circles*) as a function of *Sso*SSB concentration obtained in a background of 10 mM KCl. Values represent the average of three experiments and are given as mean ± s.e.m. *Solid lines* represent the result from a non-linear squares fit to a Hill model as described by Eq. 1. **c** Stoichiometry of the *Sso*SSB-RNA interaction was determined using tryptophan emission quenching. A 460 nM concentration of *Sso*SSB was titrated with a 12 C (*black circles*) and a 20 C (*red circles*) ssRNA oligonucleotide. The occluded site size was determined by extrapolation of the linear part of the titration curve to the point of intersection with the corresponding plateau value after saturation (*solid black lines* for 12 C and *solid red lines* for 20 C). The cross-point of the two linear fitting regimes yields, for each ssRNA, a similar value of 6–7 nucleotides interacting with each *Sso*SSb monomer. **d** *Sso*SSB induced melting of an RNA hairpin monitored using an intra-molecular FRET assay. Variation in the fraction of disrupted RNA hairpin as a function of SsoSSB concentration. FRET efficiency was calculated as described in the methods section and transformed into fraction of disrupted hairpin. The *solid line* indicates the result from a non-linear square fit to Eq. 1. *Inset* Fluorescence spectra of Cy3 and Cy5 normalized at the maximum of the Cy3 emission band (565 nm) in the absence and presence of 20 nM *Sso*SSB

To investigate this unexpected property of *Sso*SSB further, we carried out ensemble-fluorescence experiments with 12 nucleotide ssRNA and ssDNA sequences functionalized with a Cy3 dye at the 3′ end. *Sso*SSB binding to these sequences was monitored using protein-induced fluorescence enhancement (PIFE). PIFE assays are based on the increase in the fluorescence emission of dyes due to the binding of proteins in close proximity and it has been extensively used as a molecular ruler to measure binding dynamics and distances shorter than those available by Förster resonance energy transfer (FRET) (Morten et al. 2015; Lerner et al. 2016). In the PIFE assay, we replaced the 21-mer employed for ITC by 12-mer ssDNA and ssRNA strands to ensure that monomer binding is within the distance range in which the PIFE mechanism can take place. The PIFE experiments with the ssRNA strand showed a >twofold increase in Cy3 emission (Fig. 2a), similar to the increase seen previously with ssDNA (Morten et al. 2015). The binding isotherms obtained when titrating 10 nM ssRNA and ssDNA were fitted to a Hill binding model and yielded similar apparent *K*_D_ values of 4.2 ± 0.6 and 8.2 ± 0.9 nM, respectively (Fig. 2b). These values are very close to those reported previously for the binding of *Sso*SSB (Morten et al. 2015) to ssDNA under low ionic strength conditions where binding affinity is higher as demonstrated for other SSBs (Kernchen and Lipps 2006). The similarity between the affinity values also suggests that the presence of the dye at the 3′ end does not influence *Sso*SSB binding. From the fit, we obtained values for the Hill coefficients of 1.8 ± 0.3 for RNA and 1.6 ± 0.5 for DNA, implying the interaction of more than one protein with a significant degree of positive cooperativity between them. Similar values for the apparent dissociation constant (6 ± 1 nM) and the Hill coefficient (1.7 ± 0.2) were obtained when the amount of titrated ssRNA was decreased to sub-nanomolar levels (~0.7 nM).

The number of ssRNA nucleotides occluded per *Sso*SSB monomer was further investigated using the intrinsic fluorescence of tryptophan as a reporter of binding (Fig. 2c). Structural studies of *Sso*SSB have confirmed that three tryptophan residues (W56, W75 and F79) are important for ssDNA binding (Wadsworth and White 2001; Kerr et al. 2003; Gamsjaeger et al. 2015). Stoichiometric titration of *Sso*SSB (460 nM) with increasing concentrations of a 12 C ssRNA sequence induced a 75% quenching of the tryptophan emission and yielded a value of ~6–7 ribonucleotides interacting with each bound *Sso*SSB (Fig. 2c). Repeating the titration using a 20 C ssRNA yielded a similar number of nucleotides being protected by each SsoSSB monomer (Fig. 2c). This value is similar to that reported for the interaction of *Sso*SSB with ssDNA using tryptophan quenching (~5–6 nt) (Wadsworth and White 2001) and gel electrophoresis-binding assays (~5 nt) (Cubeddu and White 2005) and in general agreement with the recent SsoSSB:ssDNA NMR structure where it was shown that 5 bases are sufficient for the recognition of ssDNA (Gamsjaeger et al. 2015).

It has been shown that *Sso*SSB can melt long stretches of duplex DNA in vitro at moderate temperatures (30–40 °C) and that this melting ability is enhanced when the duplex structure contains single mismatches and lesions, such as cyclobutane pyrimidine dimers (CPD) and extra-helical adducts (Cubeddu and White 2005). To explore whether this ability to disrupt secondary structure was also present for RNA sequences, we carried out FRET experiments using a RNA oligonucleotide capable of forming a hairpin structure containing a single-nucleotide bulge (Fig. 2d). FRET has extensively been used as a molecular ruler to monitor conformational changes within proteins and DNA–protein interactions (Blouin et al. 2009). The RNA hairpin was labelled with a Cy3–Cy5 FRET pair and the change in end-to-end distance was investigated as a function of added protein (Fig. 2d). In the absence of *Sso*SSB, the fluorescence spectra obtained when exciting the Cy3 donor (*λ*_exc_ ~ 547 nm) showed a significant emission from the Cy5 acceptor dye (*λ*_em_ ~ 670 nm), indicative, as expected, of a high degree of energy transfer from the Cy3 to the Cy5 for the intact hairpin (Fig. 2d). However, in the presence of 20 nM *Sso*SSB, the spectrum was dominated by the emission from the Cy3, suggesting that the inter-dye distance had increased and, as a result, the FRET efficiency had decreased substantially. We interpreted this as evidence that *Sso*SSB can efficiently disrupt the secondary structure of the hairpin RNA as previously observed for duplex DNA (Cubeddu and White 2005).

We have recently characterized the binding dynamics of *Sso*SSB monomers to surface-immobilized ssDNA using a single-molecule FRET approach (Morten et al. 2015). Single-molecule techniques are emerging as unique tools to unravel the dynamics of protein–DNA interactions (Morten et al. 2015; Blouin et al. 2009; Craggs et al. 2014) and they have been used extensively to investigate single-strand binding proteins, such as *Eco*SSB and RPA (Zhou and Ha 2012). To compared the dynamic properties of *Sso*SSB monomers binding to ssRNA and ssDNA, *Sso*SSB was labelled with an Alexa647 acceptor dye and a 12 C ssRNA or a 12 C ssDNA was doubly labelled with a biotin group at the 5′ end for surface immobilization to streptavidin coated microscope slides and with a Cy3 FRET donor at the 3′ end.

**Fig. 3:**
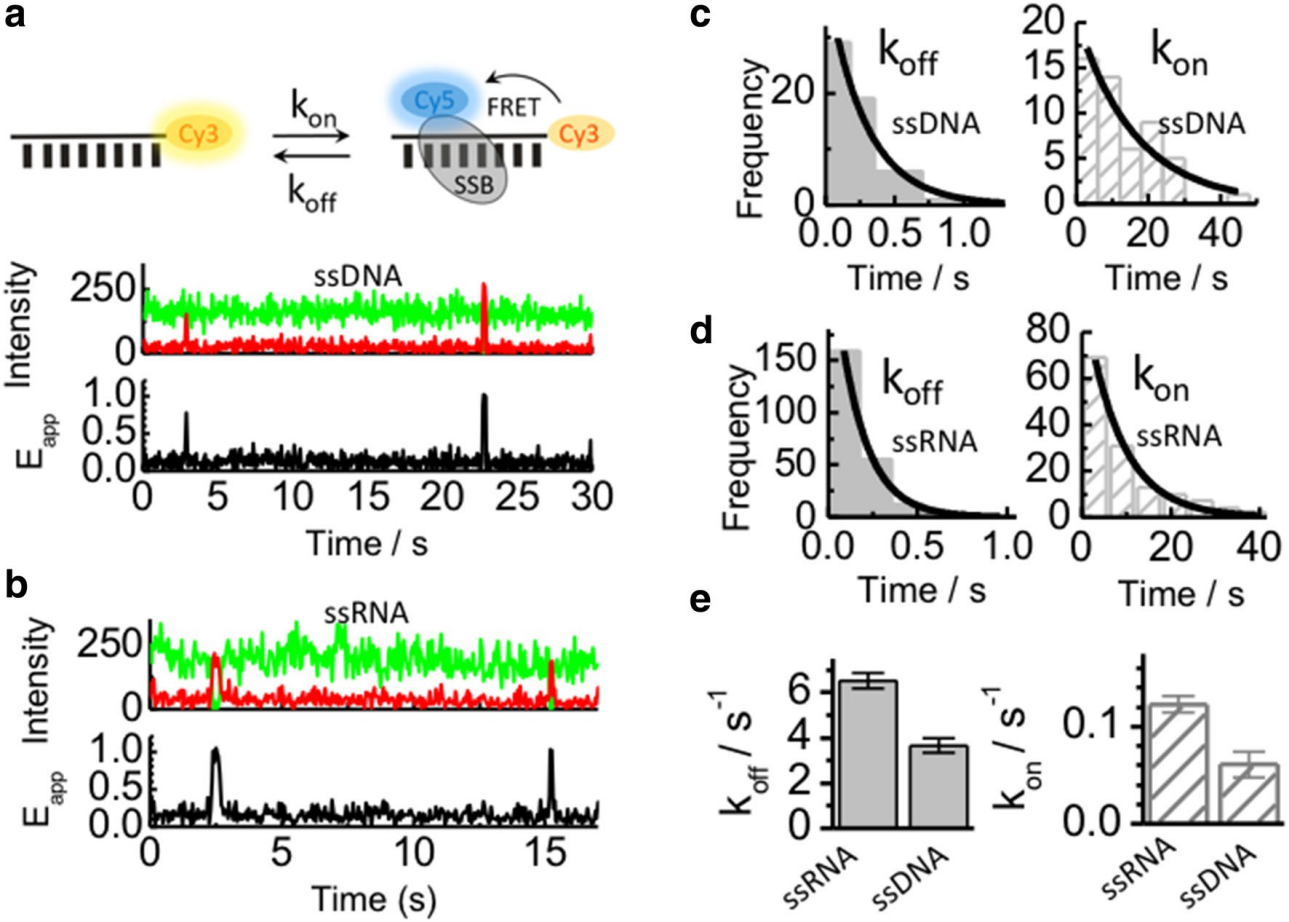
Single-molecule comparison of the interaction between Alexa647 labelled *Sso*SSB monomers and surface-immobilized 12-mer ssDNA (**a**) and 12 mer ssRNA (**b**) labelled with Cy3. Single-molecule donor (*green*) and acceptor (*red*) intensity trajectories (*upper panel*) are shown together with the corresponding FRET trace (*black, bottom panel*) obtained in the presence of 1 nM concentration of *Sso*SSB. Anti-correlated fluctuations in the Cy3 and Alexa647 intensity signals result in FRET burst that indicate *Sso*SSB association and dissociation events. Single-molecule dwell-time histograms obtained for the association and dissociation of *Sso*SSB to ssDNA (**c**) and ssRNA (**d**) are also shown. Each histogram was built from >300 events and fitted to a monoexponential decay function to extract the corresponding rate. *Bar plots* showing a comparison of the dissociation (**e**) and association (**d**) rate constants in s^− 1^ obtained for the binding of 1 nM SsoSSB to an equivalent 12-mer ssDNA and ssRNA

Representative single-molecule FRET trajectories obtained for ssDNA and ssRNA in the presence of labelled *Sso*SSB are shown in Fig. 3a, b, respectively. The single-molecule traces showed a very similar behaviour for both strands and they are characterized by sudden and short-lived anti-correlated fluctuations in the intensity signal of the Cy3 and the Cy5 dyes (Fig. 3a, b). The intensity-based trajectories were transformed into FRET traces using *E*_app_ = *I*_acc_/*(I*_acc_ + *I*_don_ *)*, where *I*_acc_ represents the intensity of the acceptor and *I*_don_ the intensity of the donor. The single-molecule FRET trajectories displayed occasional bursts in FRET efficiency from a value near zero to a very high efficiency value (*E*_app_ ~ 0.9–1). These bursts represent binding events where the association of the labelled *Sso*SSB brings the acceptor in close proximity to the donor resulting in a high FRET efficiency. The average dwell time of these binding events is similar between ssDNA (Fig. 3a) and ssRNA (Fig. 3b). We have previously demonstrated using the interaction of *Sso*SSB with ssDNA that these FRET bursts are not caused by acceptor photobleaching (Morten et al. 2015). The average photobleaching dwell time of the Cy5 dye was reported as being ~50-fold longer (~55 s) than the average dwell time of individual bursts (~1 s) (Morten et al. 2015). In this experiment, we have maintained the concentration of labelled *Sso*SSB sufficiently low (~1–2 nM) to ensure only a single *Sso*SSB associates to the nucleic acid and thus allow a direct comparison of the monomer-binding dynamics to ssDNA and ssRNA. Single-molecule dwell-time histograms quantifying the association and dissociation dynamics of *Sso*SSB to ssDNA and ssRNA are shown in Fig. 3c, d, respectively. The kinetic rate values for binding and dissociation were extracted by fitting these histograms to monoexponential decay functions. *Sso*SSB monomers exhibited, at this concentration, dissociation rate values of 3.8 ± 0.8 s^− 1^ for ssDNA and 6 ± 1 s^− 1^ for ssRNA (Fig. 3e). The association rates were much slower than the dissociation rates, with values of 0.06 ± 0.01 s^− 1^ for ssDNA and 0.12 ± 0.08 s^− 1^ for ssRNA. Overall, the single-molecule data confirm that *Sso*SSB can bind ssDNA and ssRNA with similar efficiency and that individual *Sso*SSB monomers do not indefinitely persist on either of these oligonucleotides. Considering the harsh conditions to which thermophile organisms are exposed, a highly dynamic interaction between *Sso*SSB monomers and the nucleic acid sequence may provide the optimal balance to ensure efficient protection whilst enabling access to nucleic acid processing proteins.

**Fig. 4:**
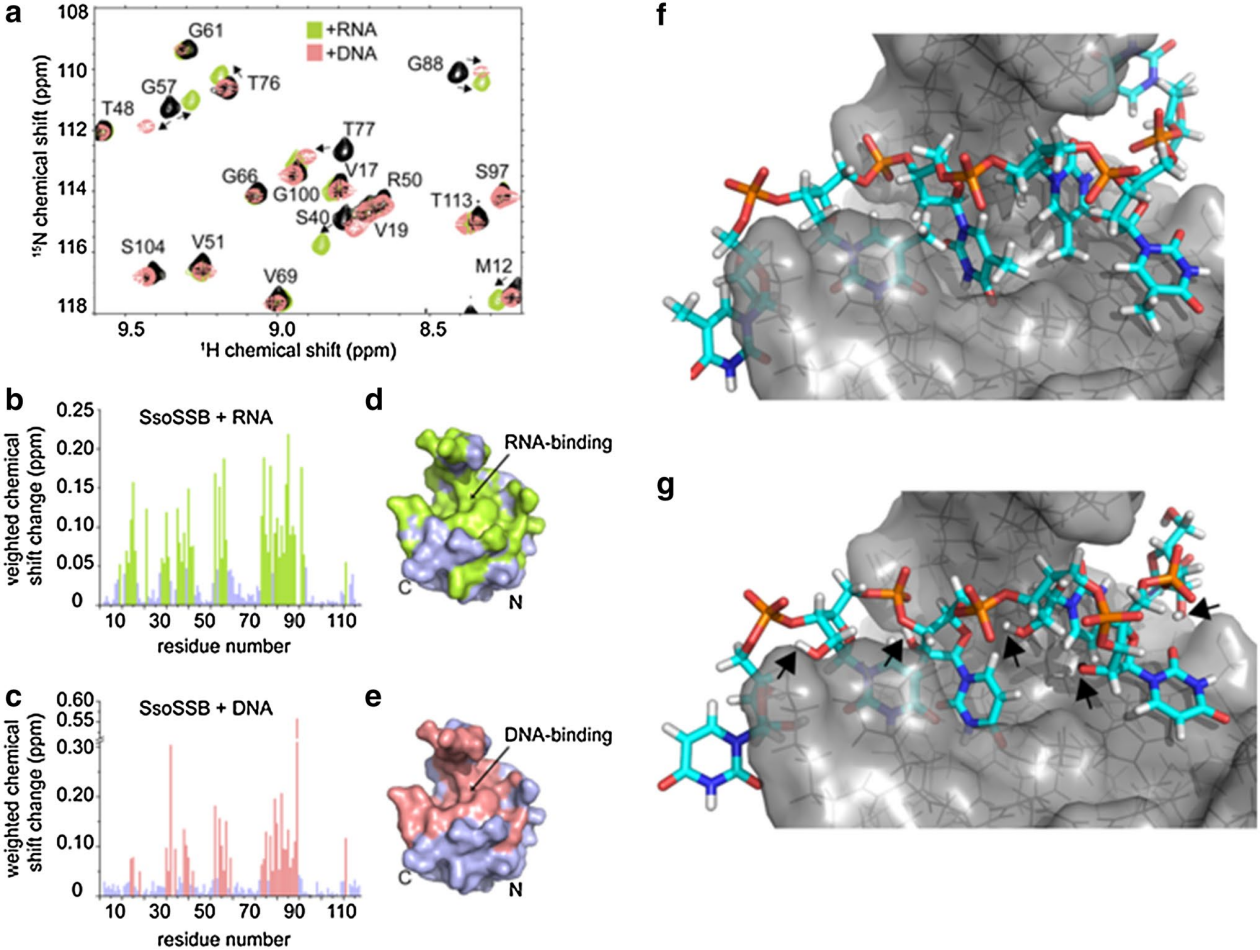
NMR and molecular modelling characterization of *Sso*SSB binding to ssRNA and ssDNA. **a** Section of a 15 N HSQC spectrum of ~0.8–1 mM SsoSSB alone (*black*) and a 1:1 mixture of *Sso*SSB with 6U ssRNA (*green*) as well 6T ssDNA (*salmon*). Assignments and directions of movement are indicated. Weighted backbone chemical shift changes of HN and N for *Sso*SSB upon binding to ssRNA (**b**) and ssDNA (**c**), respectively. Residues exhibiting changes larger than the average (binding residues) are coloured in *green* for RNA (**b**) and *salmon* for DNA (**c**). Space-filling representation of the crystal structure of *Sso*SSB (PDB 1O7I) with binding residues coloured in *green* for RNA (**d**) and *salmon* for DNA (**e**). Note the high similarity of the binding site for RNA compared to DNA. **f** Energy-lowest NMR structure (PDB ID 2MNA) of *Sso*SSB-DNA complex structure. **g** Model of *Sso*SSB-RNA structure based on DNA-bound structure. The location of the 2′ hydroxyl groups is indicated by *black arrows*

In the literature, there are examples of proteins that discriminate between ssDNA and ssRNA (Dickey et al. 2013). *Schizosaccharomyces pombe* Pot1 is the most extensively studied example of a protein that can selectively bind to ssDNA and it achieves this in a number of ways, including preferentially binding to thymine rather than uracil (Nandakumar et al. 2010). A strong hydrophobic interaction is formed between the deoxythymine and a protein binding site. In contrast, uracil lacks a methyl group, producing an energetically unfavourable gap between the RNA and protein, weakening the strength of binding to RNA. Steric clashes between the 2′ hydroxyl group with Pot1 residue Ser123 and a phosphate group on the neighbouring nucleotide have also been identified as barriers to any strong affinity between RNA and the OB fold, and so facilitate the selective binding of ssDNA (Nandakumar et al. 2010). The molecular basis for discrimination by RPA and *Eco*SSB between ssDNA and RNA is less well studied, but presumably arises from similar energetic penalties for the accommodation of the extra 2′ hydroxyl group in the binding site of the protein, or from differences in the conformational flexibility of DNA and RNA (Chen et al. 2012). Having established that *Sso*SSB binds ssRNA with a similar affinity and similar kinetics as ssDNA, we next sought to determine whether there are any major structural differences between DNA and RNA recognition. We carried out NMR HSQC experiments of ^15^N-*Sso*SSB in the absence and presence of RNA revealing that the same residues that exhibit chemical shift changes upon binding of ssDNA are also significantly perturbed when RNA is added (Fig. 4a). These data suggest that the interaction surface is conserved between ssDNA and RNA. Indeed, mapping of the observed chemical shift changes onto the crystal structure of *Sso*SSB (PDB ID 1O7I) confirmed that ssDNA and RNA recognise essentially the same binding interface on the protein (Fig. 4b–e). We have recently solved the structure of *Sso*SSB bound to ssDNA and have shown that the defining feature of the complex structure is the base-stacking of three aromatic residues (W56, W75 and F79) with three ssDNA bases (PDB ID 2MNA) (Gamsjaeger et al. 2015). The NMR data suggest that this base-stacking mechanism is conserved between ssDNA and RNA. An in-silico model (Fig. 4f–g), calculated based on the NMR structure of the DNA-bound SsoSSB (Gamsjaeger et al. 2015) (assuming that replacing the ssDNA by RNA does not lead to a major change in the conformation of the nucleotide), provides further strong support for this notion. As seen from Fig. 4g, the model demonstrates that SsoSSB’s OB fold is capable of accommodating the 2′ hydroxyl group of the RNA and the effects of the resulting ring puckering without disrupting the aromatic stacking between the bases and aromatic residues in the OB fold.

**Fig. 5:**
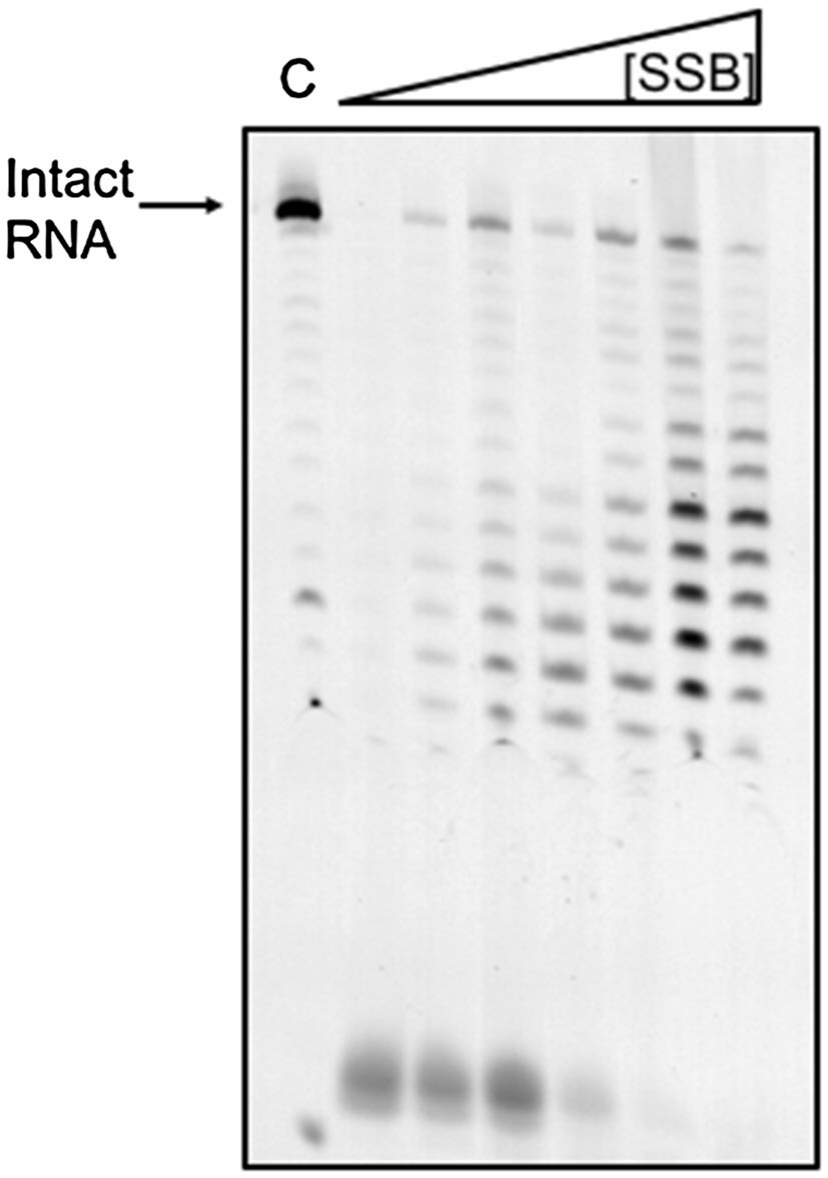
*Sso*SSB protects RNA against degradation by the archaeal exosome. A 25 nt RNA oligonucleotide labelled with FAM was fully digested by the archaeal exosome in the absence of *Sso*SSB, but exosome function was progressively inhibited when the concentration of SsoSSB was progressively increased (0, 10, 120, 240, 360, 420, and 480 µM). *Lane C* shows the undigested RNA oligonucleotide in the absence of both the exosome and *Sso*SSB

*In vivo*, RNA in *S. solfataricus* is turned over by the exosome, which functions like the eukaryotic exosome by degrading RNA in a 3′–5′ direction (Evguenieva-Hackenberg et al. 2003). We, therefore, examined the effect of *Sso*SSB on the efficiency of RNA degradation by the exosome *in vitro* (Fig. 5). A 25 nt RNA oligonucleotide labelled with a 5′-fluorescein moiety was incubated with purified *S. solfataricus* exosome in the presence of increasing amounts of *Sso*SSB. At higher concentrations of *Sso*SSB, the activity of the exosome was progressively diminished, demonstrating that *Sso*SSB has the ability to bind and protect RNA against degradative enzymes in vitro. Partial protection of RNA by *Sso*SSB against digestion by benzonase was reported previously (Shi et al. 2013).

*Sso*SSB is clearly the major ssDNA-binding protein present in *Sulfolobus* cell extracts, and is estimated to constitute 0.1% of total soluble protein (Wadsworth and White 2001; Paytubi et al. 2012). Our data suggest that *Sso*SSB has the potential to associate with and stabilise unstructured RNA molecules, such as mRNA, and thus increase its half-life at the elevated temperatures characteristic of hyperthermophilic organisms. In *S. solfataricus*, mRNA half-lives are longer than those seen in bacteria, which may reflect the increased stability and protection provided by RNA-binding proteins (Bini et al. 2002). It is also possible that *Sso*SSB plays a role in RNA remodelling in conjunction with RNA helicases, for example in ribosome biogenesis, as SSB binding could protect unfolded rRNA and act as an RNA chaperone. We have shown previously that *Sso*SSB forms a tight physical interaction with RNA polymerase via the C-terminal tail, and can stimulate transcription in vitro, consistent with a role as an mRNA chaperone (Richard et al. 2004).

There is a good reason to suppose that the OB fold evolved originally as an RNA-binding module, as RNA is thought to have predated DNA early in evolution (Orgel 1998), and several examples of OB fold domains specialised for RNA binding have been reported. Examples include bacterial tRNA-binding proteins proposed to act as molecular chaperones to protect and stabilise tRNAs (Orgel 1998), N-terminal anti-codon binding domains of some class II tRNA synthetases (Swairjo et al. 2000), translation initiation factors, and ribosomal proteins from bacteria and archaea (Li and Hoffman 2001; Wu et al. 2003). The archaeal chromatin protein Alba, whose primary role is thought to require binding to dsDNA, has been shown to also interact quite strongly with RNA in vitro (Guo et al. 2003). SSBs from several hyperthermophilic species have been shown capable of binding RNA in vitro (Shi et al. 2013). The relaxed specificity of abundant nucleic acid binding proteins in hyperthermophiles may thus be a derived feature that has evolved to protect both ssDNA and RNA under extreme conditions, or alternatively reflect an ancestral state held over from the RNA world.
